# A Survey on Applications of Artificial Intelligence for Pre-Parametric Project Cost and Soil Shear-Strength Estimation in Construction and Geotechnical Engineering

**DOI:** 10.3390/s21020463

**Published:** 2021-01-11

**Authors:** Sparsh Sharma, Suhaib Ahmed, Mohd Naseem, Waleed S. Alnumay, Saurabh Singh, Gi Hwan Cho

**Affiliations:** 1 Department of Computer Science & Engineering, BGSB University, Rajouri 185234, Jammu and Kashmir, India; sparsh@bgsbu.ac.in (S.S.); mohdnaseem@bgsbu.ac.in (M.N.); 2 Department of Electronics & Communication Engineering, BGSB University, Rajouri 185234, Jammu and Kashmir, India; suhaib@bgsbu.ac.in; 3 Computer Science Department, King Saud University, Riyadh 11451, Saudi Arabia; wnumay@ksu.edu.sa; 4 Department of Industrial & Systems Engineering, Dongguk University, Seoul 04620, Korea; 5 Division of Computer Science & Engineering, Jeonbuk National University, Jeonju 54896, Korea

**Keywords:** artificial intelligence, artificial neural network (ANN), construction engineering, geotechnical engineering, IoT, pre-parametric cost, project duration, shear strength of soil, support vector machine (SVM)

## Abstract

Ensuring soil strength, as well as preliminary construction cost and duration prediction, is a very crucial and preliminary aspect of any construction project. Similarly, building strong structures is very important in geotechnical engineering to ensure the bearing capability of structures against external forces. Hence, in this first-of-its-kind state-of-the-art review, the capability of various artificial intelligence (AI)-based models toward accurate prediction and estimation of preliminary construction cost, duration, and shear strength is explored. Initially, background regarding the revolutionary AI technology along with its different models suited for geotechnical and construction engineering is presented. Various existing works in the literature on the usage of AI-based models for the abovementioned applications of construction and maintenance are presented along with their advantages, limitations, and future work. Through analysis, various crucial input parameters with great impact on the estimation of preliminary construction cost, duration, and soil shear strength are enumerated and presented. Lastly, various challenges in using AI-based models for accurate predictions in these applications, as well as factors contributing to the cost-overrun issues, are presented. This study can, thus, greatly assist civil engineers in efficiently using the capabilities of AI for solving complex and risk-sensitive tasks, and it can also be used in Internet of things (IoT) environments for automated applications such as smart structural health-monitoring systems.

## 1. Introduction

Artificial intelligence (AI) has already revolutionized various daily activities, as well as sectors such as healthcare, agriculture, transportation, and education [1,2,3]. The construction sector has also not been untouched by AI, which is expected to assist civil engineers in the automation of various construction-related tasks which are normally time-consuming and labor-intensive.

The construction of buildings, dams, highways, bridges, and roads requires substantial effort, cost, and strategies for robust, reliable, and beautiful structures. Manual constructions usually require enormous effort and cost in terms of labor, time, and holistic thinking. The use of robots for the construction of building walls can be easily seen in practice in various countries [4]. Robots, as well as augmented and virtual reality, are brought into use for performing repetitive, hazardous, and risky construction jobs such as welding, which humans are reluctant to do. The AI technology of learning from an existing knowledgebase is used to automate various civil- and geotechnical-related applications, such as the estimation of compressive strength of concrete, shear strength of soil, project pre-cost and duration, structural health monitoring, crack detection, pothole detection, and many more [5,6,7,8].

According to various studies, it is estimated that, by the year 2050, no less than two-thirds of the world’s total population will live in new cities with advanced and robust structures and roads [9]. In order to meet this expected demand, the rate of construction is required to be increased manifold. While increasing the pace of construction, the quality of structures is expected to be maintained. Thus, for achieving these tasks, AI can be brought into use, which will not only boost the construction pace but also assist the engineers in maintaining the quality of constructed buildings in a lesser amount of time with high accuracy and precision.

### 1.1. Need for AI in the Prediction of Construction Parameters

In geotechnical and construction engineering, various parameters are required to be computed beforehand for the estimation of properties such as shear strength of soil, compressive strength of concrete, total cost to be incurred in the project, and duration of the project. These properties are used for ensuring the quality, durability, reliability, robustness, and ability to withstand external forces of a structure.

For instance, the construction of good roads is one of the important aspects in providing a secure and congestion-free driving experience. For the construction of roads, various factors such as the cost, time, labor, and type of material to use need to be considered beforehand. If a developing country such as India is considered, before the construction of roads, the important parameter of cost estimate is required to be computed. However, its calculation in the construction of roads requires civil engineers and the Central Public Work Department (CPWD) to manually calculate road parameters such as slope, angle, and elevation. According to various calculations and in consultation with various CPWD personnel, it was found that approximately 15,000 INR per km is spent estimating these road parameters. This manual calculation of road pre-construction parameters is not only cost-extensive but also time-consuming, which eventually slows the overall growth of highway construction in India.

In the era of machine learning and artificial intelligence, this preliminary calculation of road construction parameters can be done automatically with the use of AI and computer vision techniques with zero to minimal human intervention. Machine calculation of parameters allows not only lowering the cost and time required but also enhancing the accuracy in their calculation by eliminating any type of human error. In Table 1, the benefits of using AI assisted technology in roads and building construction are presented.

The construction industry plays a very crucial role in boosting the economics of any developing and developed country. The time, cost, and quality of construction projects are the factors underlying the success of this sector. The estimation and prediction of pre-construction outputs such as the expected duration of a project, expected cost to be incurred in a project, shear strength of a soil, and compressive strength of concrete through the use of initially limited available parameters ensures the performance of the project in terms of quality, cost, and on-time completion.

Since it is well known that construction projects are susceptible to issues such as cost overrun, delay in completion, and damage to structures due to improper estimation of quality parameters, in order to prevent these common issues from occurring in any construction project, the prior computation and prediction of parameters such as cost, duration, and quality of soil in use need to be done.

However, the prior prediction and estimation of these project parameters are challenging tasks considering the fact that very little to no information regarding the project is available in its initial stages.

Furthermore, there exist various construction-related factors and unexpected variables that greatly affect the estimation and calculation of the abovementioned parameters. These factors and unexpected variables can be both external and internal, and they vary from project to project, making the estimation of parameter values on their basis a challenging task.

### 1.2. Main Objectives of the Study

**Figure 1:**
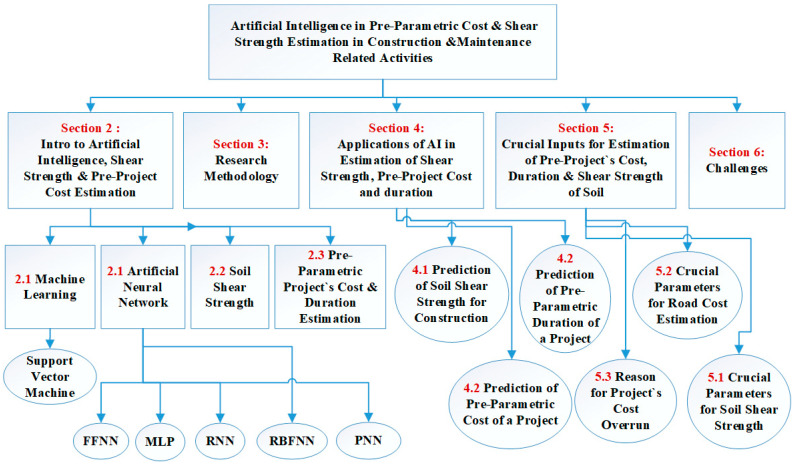
Blueprint of this paper.

The main aim of this study was to explore and highlight the role of AI in the civil engineering and construction sector. In this study, various applications of AI in civil engineering, especially focused on construction, are explored by considering articles from 2005 to the present year (2020). This article aims to assist researchers and civil engineers in determining the strength and potential of AI in the construction sector, which will eventually assist them in automating the manual, repetitive, and time-consuming tasks with high precision and lower effort and cost. Figure 1 shows the blueprint of the whole article.

## 2. Artificial Intelligence and Its Application in Shear Strength and Pre-Project Cost Estimation

### 2.1. Introduction to Artificial Intelligence

The term AI was first coined in the year 1956 and aims at emulating the performance and capabilities of humans [10]. In AI, machines learn and improve from their past experiences as humans do. Machine learning (ML) is a subset of AI in which machines are allowed to learn, react, and make decisions on the basis of their experience, i.e., input data. Similarly, deep learning [11,12,13,14,15,16,17,18,19,20,21,22] is a further subset of ML and AI.

With the improvements in the capabilities of high-performance computing technology and the comprehensive development of the use of artificial neural network (ANN)- and ML-based models, AI has seen tremendous improvement and can be easily found in various day-to-day activities in industries such as healthcare, cybersecurity, forensics, stock exchange, and Internet of things (IoT). AI has revolutionized the performance of various sectors in terms of its high efficiency and accuracy, leading to low cost and duration of projects [23].

AI supports a plethora of applications. It is used in applications and domains such as facial recognition, smart transportation systems for object detection, traffic-light monitoring and control, image recognition, malware detection, and the stock exchange.

The usage of AI and its models can also easily be seen in the domain of construction. Construction tasks usually require a high level of precision and expertise for the generation of complex, strong, and beautiful structures. Any error can lead to losses in terms of people’s lives, as well as losses in infrastructure. AI models, therefore, can be used for performing various tasks in construction and geotechnical engineering where high accuracy and precision are required.

There are various issues and challenges in civil and geotechnical engineering, especially in construction management, design, and decision-making, which are profoundly impacted by various uncertain factors that not only require mathematical, mechanics, and physics knowledge but also the experience of the practitioners. These issues and challenges cannot be handled through traditional procedures. However, these complex issues can be solved easily using AI [24].

Some applications of geotechnical and civil construction engineering where AI models can be used include (a) the estimation of soil shear strength related to its ability to bear high load and external pressure due to floods and other natural calamities, (b) the prediction of concrete compressive strength, (c) the prediction of concrete-beam shear strength [25,26,27], (d) the prediction of the shear strength of peaks [28], walls [29], rocks [30], etc., and (e) the accurate estimation of pre-project bid cost and duration with minimal risk of cost and duration overrun.

There are various methods and technologies within AI which improve efficiency.

**Figure 2:**
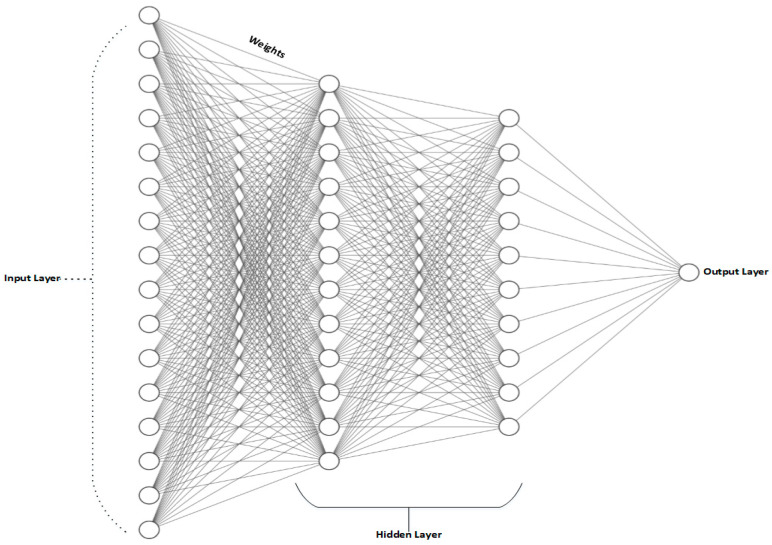
General structure of an artificial neural network (ANN).

1. **Machine learning** is a subfield of AI that grants machines the ability to learn and develop from their past experience without being explicitly programmed. Machine learning, according to the type of training provided to the model, can be broadly categorized as supervised learning, unsupervised learning, and reinforcement learning. In supervised learning, which is also called learning with teacher or guided learning, labeled data with a desired output are provided as input to the machine. However, in the case of unsupervised learning, which is also referred to as learning without a teacher, no labeled input data are provided to the machine [31,32,33,34,35,36]. The machine instead tries to draw inferences from the dataset containing unlabeled responses. There exist various machine-learning algorithms such as decision trees, regression, and random forest. However, in this article, only those algorithms previously applied in the prediction of soil shear strength and project pre-cost estimation are listed.
  - **Support vector machine (SVM)** [37] is a binary classification model, capable of generating a hyperplane to isolate data samples on the basis of maximum margin principles in order to achieve minimum structural risk [38]. There are essentially two concepts used in SVM. The first concept is the optimal margin classifier, which is a linear classifier that generates a distinct hyperplane, also termed a decision surface, such that the gap is maximized between the negative and positive instances. Kernel functions represent the second concept. A kernel function is used to compute two vector dot products. The use of efficient nonlinear mapping of the kernel to the original example data ensures that the data, which was nonseparable in the original input space, can be separated linearly into a high-dimensional functional space [39]. This allows solving nonlinear partitions through the addition of a kernel function [40].
     The SVM’s partition function is commonly used to solve pattern recognition, matter classification, filter problems, and various other problems in geotechnical engineering. The classification of soil and rock is one such research application, which allows engineers to decide the correct building materials and construction methods for ensuring safety, depending on the category of soil and rock.
     Landslides are a crucial field of research in geotechnical engineering, since they pose enormous threats to public safety and frequently lead to major property losses. SVM can be used for analyzing susceptibility to landslides in advance. One-class SVM and two-class SVM can effectively predict susceptibility to landslides even with limited data. However, two-class SVM is more sensitive to the number of samples and more accurate than its counterpart [41].
     SVM can also be used for solving regression problems, basically involving the determination of a regression model for describing the relationships among sample data. The identification of deformed rocks and soil can also be performed using SVM in geotechnical engineering.
  - **Least square support vector machine (LSSVM)** is a statistical learning technique that employs the loss function of a least square linear system [42]. LSSVM aims to reduce the computational complexity of SVM. The inequality constraints for solving quadratic problems are replaced by equality constraints in the case of LSSVM, leading to faster training speed compared to SVM. However, LSSVM’s solution suffers from a lack of robustness and sparseness. This limitation leads to an increase in the training time and reduced prediction accuracy, especially for industrial datasets, which generally contain explosions of data, imbalanced distribution, and heteroscedasticity [43]. While a single LSSVM with reconstructed input samples and optimum parameters has excellent predictive efficiency under some conditions, it may have certain kinds of inherent bias in other cases due to its fixed kernel feature [44].
2. **Artificial neural network (ANN)** is a part of artificial intelligence. The concept of ANN is not new and is inspired by the way human biological neurons work in the human brain. ANNs are quite helpful in giving optimal solutions to complex problems that cannot be analytically defined. ANN consists of fundamental processing units called neurons, along with weighted connections between them. ANN can be defined as a large, parallel dispersed data-processing network which consists of simple entities called neurons. It has a natural propensity to store experiential information which is then used, analogously to the way the brain collects and holds information [45]. During the learning process, the neural network acquires information and preserves it through the intensity of neuronal contact [46]. Neural networks are designed such that problem-solving is possible without the need for experts and without programming. In unclear data, they often search for patterns and connections and are specifically tailored for complex problems where there are no classical mathematical and conventional procedures or formal underlying theories. ANN differs from statistical and algorithmic techniques such as regression sampling in that ANN learns from examples to give generalized solutions [47,48,49,50,51,52,53,54].
   ANN consists of multiple layers, and, in every layer, there exist nonlinear processing and fundamental computation units called neurons that perform tasks such as feature extraction. The output from every layer is fed as input into the subsequent layer. ANN-based models work by collecting their input from various neurons present at the input layer, and they are designed to sense data from the outside world just as humans do, passing this information collected from different input neurons to further neurons present in another layer of hierarchy termed the hidden layer. The information is then processed at this layer and is passed to the output layer. A typical ANN structure is shown in Figure 2.

ANN completely depends upon two important phases: (i) training phase and (ii) testing phase. The training phase involves the labeling of vast volumes of data and deciding their corresponding characteristics, while the testing phase uses previous experience to draw conclusions and label the new unexposed data [55]. During the training of the ANN, a dataset is provided, and weights between the interconnections are adjusted to reach the output specified in the training dataset. If the output is known, it is deemed supervised training; otherwise, it is deemed unsupervised training. The basic elements that characterize an ANN are listed below [56].

- Topology of the network;
- Training method being employed;
- Type of association between input and output;
- Presentation of the information.

ANN offers the following advantages over AI techniques, making it a popular choice for use in a plethora of domains:

- Self-organization: ANNs are self-organizing. They can create their own structures and can adjust weights on their own as per the requirements.
- Fault tolerance: Even if some neuron is not responding, some piece of information is missing, or data are distorted and noisy, ANNs can still produce the output and have the capability to locate the fault.
- Adaptive learning: ANNs have the ability to learn on their own by choosing optimal features and weights, and they produce outputs not limited to the provided input.
- Capability to deal with large data: ANNs work equally well for large datasets. An increase in the number of training samples can help the models to improve their learning through exposure to different possible scenarios, thus improving their generalizability.
- In ANNs, the input data are stored in the network instead of a database. Thus, any loss of data has no effect on their working.
- Online and multi-task operations: ANNs can be implemented in parallel to perform multiple tasks simultaneously without hindering the performance of the system. Moreover, they are specially configured to perform online processes.

ANNs can be classified according to their architecture and the number of hidden layers present. In this section, we present some popular types of neural networks used by researchers in construction and geotechnical engineering for tasks such as shear-strength estimation and pre-parametric prediction of project cost and duration.

**Figure 3:**
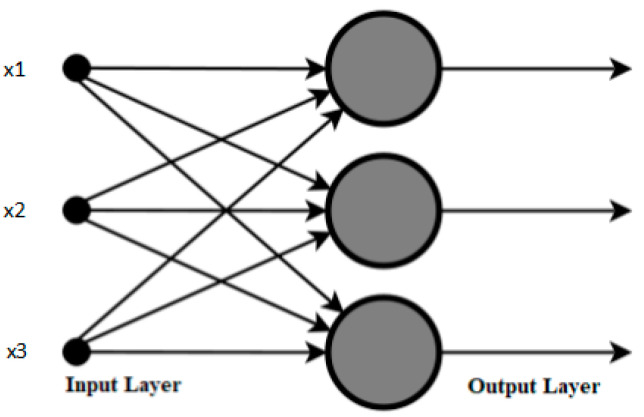
General structure of a feed-forward neural network (FFNN).

**Figure 4:**
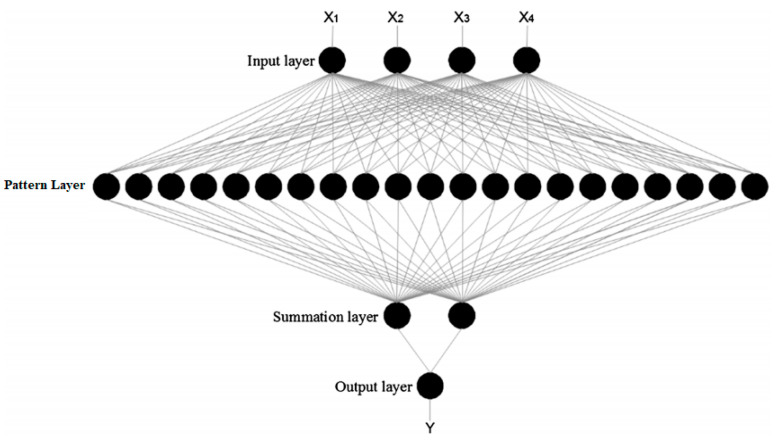
General structure of probabilistic neural network (PNN).

- **Feed-forward neural network (FFNN)** is the simplest and most basic type of neural network and may or may not feature a hidden layer. Information in FFNN flows in only one direction (forward propagation), i.e., from the input layer to the processing hidden layer to the output layer [57,58]. Figure 3 shows the basic structure of an FFNN.
- **Multilayer perceptron (MLP)** is a simple and commonly used neural network which consists of one input layer, one or more hidden layers, and one output layer. MLP is mostly used for problem-solving where learning is performed through backpropagation [59]. This network propagates the data from the input to output layer through the network and detects errors; then, by incorporating it into the learning formula, it propagates the data back to the input layer. Gradient descent optimization is used for reducing the error between the actual desired output and the predicted output by reupdating the weights of the neurons.
- **Recurrent neural network (RNN)**, also called long short-term memory (LSTM), is the most widely used and most complex type of neural network, in which the information flows bidirectionally. The output of the processing nodes is stored in this network and is used for improving their performance. RNN works by saving the output from a layer and feeding it back to the input to help predict layer outputs. The first layer is formed as a result of the sum of weights with characteristics similar to the feed-forward neural network. Once this is determined, the RNN phase starts in which each neuron in the subsequent time step recalls any information it had in the preceding time step. This allows each neuron to act as a memory cell when conducting computations. RNN works via forward propagation and remembers the information it needs for later use. Whenever the prediction is incorrect, the learning rate or error correction is used to make minor adjustments in order to improve the model’s prediction through backpropagation [60].
- **Radial basis function neural network (RBFNN)** is a type of multilayer ANN consisting of an input, hidden, and output layer. RBFNN’s hidden layer consists of hidden neurons, which are activated by the Gaussian function. Training of the RBFNN is split into two phases. Firstly, weights are calculated from the input to the hidden layer, and then weights from the hidden to the output layer are determined. Because of its compact topology and fast learning speed, RBFNN has attracted extensive attention compared to other neural networks, and it has been widely used in many research and engineering sectors [61,62].
- **Probabilistic neural network (PNN)** is basically a classifier. Unlike other backpropagation-based ANNs, PNN is based on the kernel discriminant analysis (KDA), which is a statistical algorithm in which the operations are structured into a multilayered feed-forward network. The interest in pattern recognition using PNN is growing due to its unique quality of interpretation using the probability density function. PNN has many benefits over well-known backpropagation (BP)-based ANNs. The biggest benefit that PNN offers over other neural networks is that training can be completed quickly and effectively. Unlike BP networks, weights are assigned and not trained. Therefore, the original weights always remain the same, and only the new vectors are introduced into weight matrices during the training process. Therefore, the operation happens in real time, and the network classifies input vectors into a specific class. The PNN consists of an input layer, pattern layer, summation layer, and output layer, as shown in Figure 4. The first layer, which is the input layer, takes the input and is completely connected to the pattern layers such that every neuron of the input layer has a connection with all the neurons of the pattern layer. Weight values in this layer are set equivalent to the different training patterns. In the summation layer, summation neurons compute the probability density function. Every neuron of the summation layer adds outputs of the pattern layer neurons, which basically corresponds to the class from which the training pattern is selected [63,64].

### 2.2. Soil Shear Strength

In geotechnical and civil engineering, soil shear strength is a very important parameter that is used and estimated during the construction of structures such as dams, pavements, and retaining walls. Internal friction and cohesion are the parameters that define the shear strength of a soil sample. The shear strength of a soil determines its capability to bear slippage and internal movement when exposed to some load. Shear strength, thus, determines the withstanding capability of an infrastructure. The shear strength of a soil sample is affected by various factors such as the liquid limit, plastic index, and moisture content, and it can be computed in laboratories. However, the process of computing the soil shear strength in laboratories is not only time-consuming but costly also owing to difficulties in the handling of instruments, as well as the long measurement procedures for ensuring reliable and accurate results. Therefore, AI can be used for the accurate and timely computation of soil shear strength.

The general steps involved in the estimation of soil shear strength through AI are as follows:

- Problem identification;
- Data collection and preprocessing of the database;
- Identification of crucial input parameters;
- Selection of AI-based prediction model;
- Performance comparison of developed AI models;
- Sensitivity analysis;
- Prediction based upon the output of the best AI model.

### 2.3. Pre-Project Cost and Duration

Project cost and duration play a very important role in construction. Prior information about the cost that will be incurred throughout the construction project, along with the time it will take to complete the project, can be beneficial for both constructors and clients. The prediction of project cost can help bidders to make a suitable bid after including their profits and margins, as well as prevent the issue of cost overrun, which is usually seen in construction projects. However, accurate pre-project prediction of cost and duration requires certain information in the form of input parameters, which are limited in nature. This pre-project estimation of cost and duration can easily be computed using AI models, with steps similar to those involved in the prediction of soil shear strength.

## 3. Research Methodology

The revolution that AI has brought about in various sectors is easily visible, and it has greatly improved the efficiency, productivity, security, cost, and labor burden in different work areas. The applicability of AI in the construction sector is also remarkable, leading to various improvements in construction-related activities. For instance, with AI, construction-related tasks have been automated. The burdens of cost, labor, and time have been greatly minimized. Tasks where human lives are at risk and which humans are reluctant to do have been shifted to AI. The pace of construction has also increased manifold, and beautiful and complex structures are being developed with great ease. AI is also used for the pre-project estimation of cost and duration with minimal information available. This prior estimation of construction cost and duration can help contractors in setting the most optimal bids for a project after including their profits, thus avoiding the problems of delay and cost overrun during construction. Similarly, AI can be used for automating the process of soil shear-strength estimation using various crucial input soil parameters, thus enabling the development of strong structures that can withstand natural disasters such as earthquakes and floods.

In this study, an attempt was made to highlight the importance and applicability of AI-based models for performing automatic calculations and predictions of crucial parameters such as soil shear strength and project cost and duration, which are important aspects of construction, using the minimal information available.

The importance of automatic predictions and calculations of cost, time, and shear strength parameters was discussed in the previous section. AI-based models can definitely solve various problems in the process of construction, such as cost overrun, delays, and building collapse. AI models make use of various factors and parameters which play an important role in the calculation and prediction of the abovementioned parameters. In fact, there exist a plethora of AI-based algorithms and models used by researchers across the globe for solving problems in construction-related activities [65].

This motivated us to highlight various relevant studies available in the literature using various AI-based techniques in making accurate and low-cost predictions of important and crucial parameters outlined previously.

In this first-of-its-kind state-of-the-art survey, an attempt was made to examine the existing work of various researchers focused on using AI techniques for the estimation, prediction, and calculation of crucial parameters in construction-related jobs. The methodology followed in performing this study was as follows:

- Initially, various AI-based models and algorithms widely used in the geotechnical field by researchers in the literature were studied and analyzed to identify their pros and cons.
- The various areas of civil construction and maintenance where AI models can be applied were explored in detail. This was done by referencing available relevant research articles over the past decade that were published in reputed peer-reviewed journals, conferences, book chapters, etc.
- Important input parameters with an important role in and impact on the prediction and estimation of cost, time, and shear strength are presented.
- Existing challenges, research gaps, and future research directions are presented at the end of the article with the expectation of providing a path to help researchers already working in this domain.

**Figure 5:**
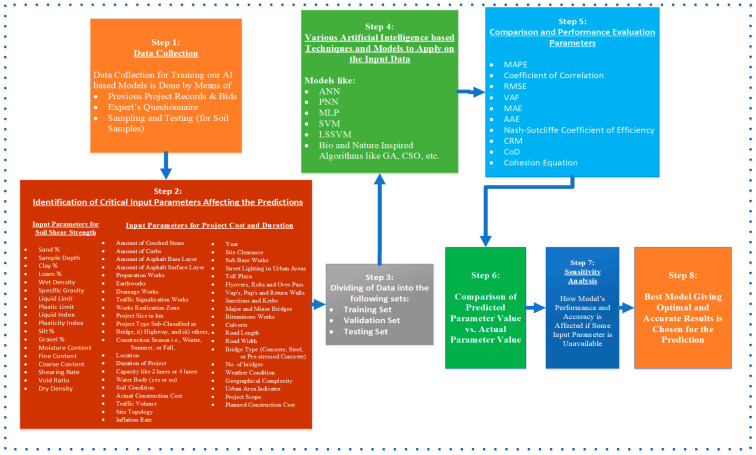
Basic steps involved in the use of AI-based models in the prediction and estimation of shear strength and pre-project cost and duration throughout the construction of roads.

Figure 5 shows the methodology and steps involved in the prediction of cost, duration, and shear-strength parameters throughout a construction project.

## 4. Applications of Artificial Intelligence in Civil Construction and Maintenance-Related Tasks

Good-quality structures such as dams, roads, highways, buildings, and bridges are key to a country’s development. In this section, the existing work of various researchers related to the use of AI in the Prediction of soil shear strength and pre-project cost and duration is discussed in detail.

### 4.1. Predicting Soil Shear Strength for Construction

It is very important to determine the soil shear strength before doing any type of construction. Shear strength is defined as the ability of soils to withstand internal movement or slippage when subjected to an imposed load. This property is a key element that is often used in the planning and design of many large-scale infrastructure projects, including high-rise buildings, roads, sidewalks, earth dams, and walls.

Shear strength is the property of soil that helps it to maintain balance in situations when the ground surface is not level or if the load is intense, which can cause shear stress. A plethora of methods and techniques have been proposed in the literature for determining the shear-strength parameters of unsaturated soil. Such shear parameters can be measured in the field and/or in the laboratory. However, the method of obtaining soil shear strength in the lab is not only expensive but also time-consuming. Hence, a new robust model to accurately predict futuristic shear strength is highly desirable, such that slow and cumbersome laboratory research can be avoided.

Soft computational methods such as ANN, fuzzy techniques, and genetic algorithms have been used to solve a wide range of geoscience and geotechnical engineering problems such as landslide susceptibility zonation (LSZ), debris flow prediction, landslide monitoring, and rock strength prediction. AI can be used for the estimation of soil shear strength by considering various factors such as clay content, moisture content, and liquid limit as input.

**Figure 6:**
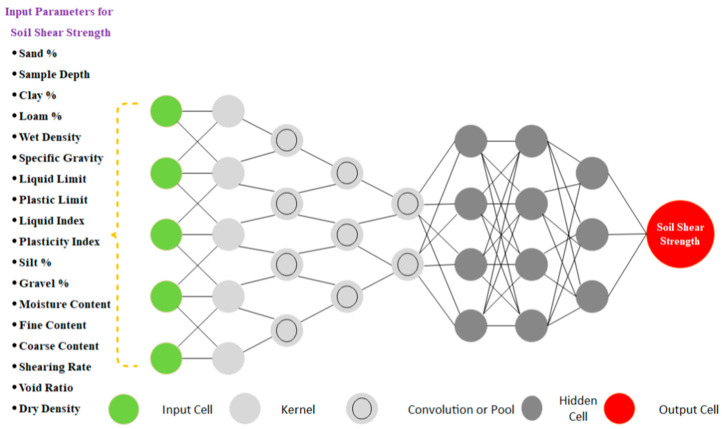
Typical ANN structure for the estimation of soil shear strength.

AI-based approaches are very useful in nonlinear modeling and can take several input variables into account when determining soil shear strength. AI models are also flexible and can adjust their structures according to changes in the collected geotechnical data [66]. A general ANN-based model depicting the use of various input parameters for the prediction of soil shear strength is shown in Figure 6.

**Figure 7:**
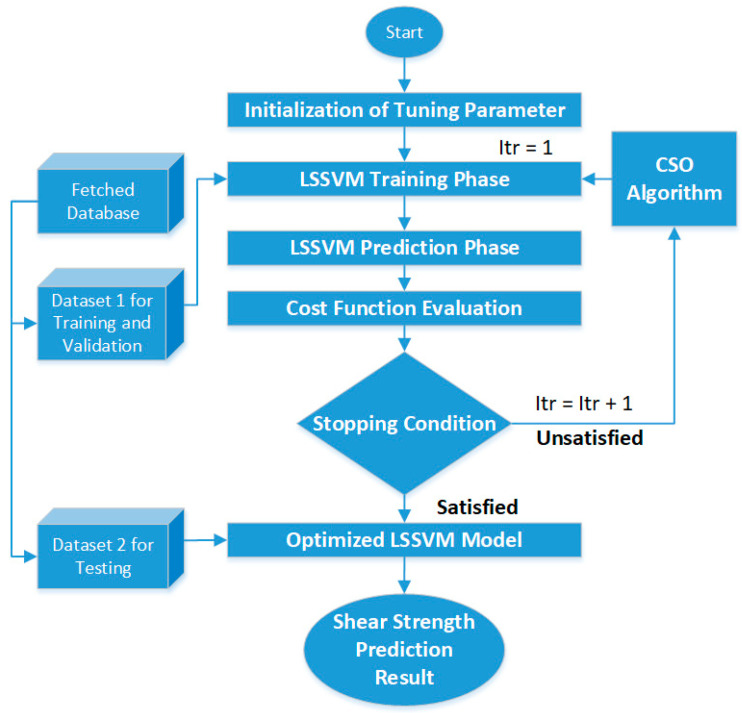
Working of least square support vector machine (LSSVM) model for shear strength prediction.

The authors in [66] made use of the swarm intelligence-based machine-learning technique for the prediction of soil shear strength during road construction. The AI technique used for this parameter calculation was a hybrid of two AI techniques: (i) least square support vector machine (LSSVM) and (ii) cuckoo search optimization (CSO). In this work, a dataset containing 332 soil samples gathered from the Trung Luong National Expressway Project in Vietnam was used for training and validation of the model. For predicting the shear strength, input parameters such as the sand percentage, sample depth, percentage of clay and loam, specific gravity, wet density of soil, plastic limit, liquid limit, liquid index, and plastic index were used. In this hybrid AI model for predicting soil shear strength, LSSVM was used for the task of generalizing the functional mapping that predicts the shear strength from the abovementioned input parameters. Since LSSVM requires the proper setting of kernel function and regularization parameters, CSO was used for their automatic computation. The flow of the proposed LSSVM and CSO algorithm is shown in Figure 7. From the experimental and simulation results, it was found that the proposed hybrid AI model using LSSVM and CSO was able to outperform other benchmark approaches including standard LSSVM, ANN, and regression trees, thus showing that AI can be used to facilitate civil construction jobs. The complete model was coded, implemented, and tested using the MATLAB software.

**Figure 8:**
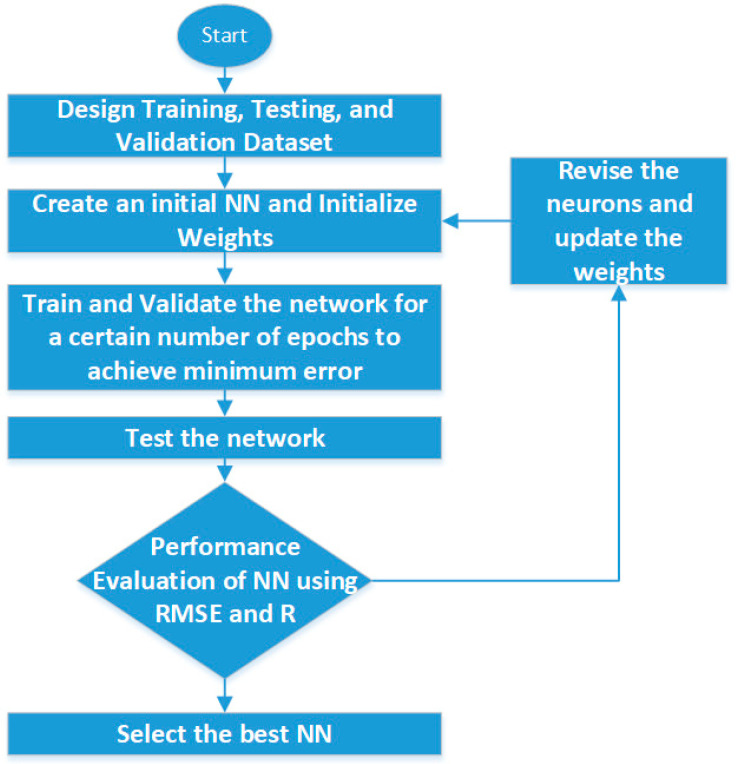
Flow of ANN model for the prediction of soil shear strength.

Similarly, the authors in [67] used a regression tree and ANN for the computation of soil shear-strength parameters such as cohesion and angle of internal friction. Six input parameters (namely, sand, silt percentage, gravel percentage, clay percentage, plastic index, and dry density) were used and fed as input to the ANN and regression tree-based model for the prediction of soil shear strength. A total of 115 soil samples were considered, of which 90 were used for training, while the remaining 25 were used for testing the performance of the model. The correlation coefficient (*R*) and root-mean-square error (RMSE) were evaluated for both the regression tree and the ANN during the performance evaluation, and it was observed that, for the prediction of internal friction angle, both techniques performed equally well, whereas, for the estimation of cohesion, the ANN outperformed the regression technique. Furthermore, all six input parameters considered were found to be important for the estimation of shear parameters. MATLAB software was used for the coding and implementation of the model. The flow of the proposed ANN model for the calculation of shear-strength parameters is shown in Figure 8.

For the estimation of soil shear-strength parameters, the authors in [64] made use of PNN, in which input parameters such as water content, dry density of soil, and the percentage of gravel, sand, silt, and clay in soil were considered. Two soil shear-strength parameters, cohesion and internal friction, were predicted using the neural network in this work. A total of 300 soil samples from 20 different boreholes in Ranchi, Jharkhand, India were collected and used in this work. The PNN model in this work had four layers, namely, (i) input layer, (ii) pattern layer, (iii) summation layer, and (iv) output layer, as shown in Figure 3. The input layer of the proposed model consisted of one neuron that fetched one set of new input data, i.e., the test data. Similarly, the pattern layer comprised 300 neurons, and each neuron had one set of input data with information on the seven soil parameters. The summation layer consisted of 16 neurons and represented each class. In the output layer, there was one neuron which output the best class. On the basis of the results, the authors concluded that neural models gave better results than mathematical models.

Similarly, for the prediction of soil shear strength, the authors in [68] made use of a functional network. The database for training the proposed predictive model was taken from [69], which consisted of a total of 131 soil samples from landslide areas, slope failure areas, volcanic eruption areas, etc. Input parameters that were considered for soil shear-strength estimation included liquid limit, plasticity index, and clay fraction. In order to compute the performance and efficiency of the proposed functional network-based predictive model, the authors compared their model with SVM- and ANN-based models using performance metrics such as the Nash–Sutcliffe coefficient of efficiency, correlation coefficient, maximum average error (MAE), RMSE, and absolute average error (AAE). According to the results, it was observed that the author’s proposed functional network-based model performed better than the ANN in terms of the Nash–Sutcliffe coefficient of efficiency and correlation coefficient. However, its performance was not found to be better in comparison to the SVM-based model.

The authors in [69] demonstrated the use of different variations of ANN and SVM models for the calculation of soil residual strength by applying these different machine-learning techniques to different input soil parameters such as the clay fraction, liquid limit, and plasticity index. A total of 137 samples for testing were taken from available databases describing samples from different areas such as landslide areas, volcanic eruption areas, debris flow areas, and slope failure areas. Out of the 137 available samples, 70% (96 samples) were used for training, while the remaining 30% (41 samples) were used for testing the performance of the models. In this study, the authors designed two ANN models for training, namely, the differential evolution neural network (DENN) and Bayesian regularization neural network (BRNN), which were compared with the Levenberg–Marquardt neural network (LMNN). Different combinations of the input parameters were considered in the application of the abovementioned ANN models. From the results, it was found that the model in which all input parameters were considered produced better results in terms of the correlation coefficient. This study by the authors suggested the possibility and success of using ANN models for different soils of different origins.

For the construction of robust houses, the estimation of soil shear strength is crucial. The authors in [70] hybridized AI-based support vector regression (SVR) and particle swarm optimization (PSO) for the prediction of soil shear strength. A set of 12 input parameters, namely, plastic limit, moisture content, void ratio, content of sand, liquid index, plastic index, dry density, liquid limit, loam content, sample depth, wet density, and clay content, were fed to the AI-based model for prediction. A dataset containing 443 samples of soil collected from a Vietnam housing project was used for the training and validation of model performance. In this hybrid AI model, SVR was used as the function optimization method for providing the mapping to predict the soil shear strength using the considered input parameters. PSO was used for optimizing the training phase of the SVR’s function approximator. For the evaluation of model performance, metrics such as MAPE, RMSE, and *R^2^* were used, and it was observed that the author’s proposed hybrid AI-based model consisting of SVR and PSO yielded better prediction accuracy in terms of RMSE, MAPE, and *R^2^* with values of 0.038, 9.701%, and 0.888, respectively.

In Table 2, other studies available in the literature for the prediction of soil shear strength using AI are presented in detail.

#### Discussion

According to the above analysis of the literature, a general observation is that artificial-intelligence-based techniques are efficient and highly useful for soil shear-strength prediction. Furthermore, as the topic of concern is complex, other advanced and hybrid AI models, along with the hyperparameter tuning, can be explored to enhance the performance of the existing models.

### 4.2. Prediction of Road Building Cost and Project Duration

Cost estimation is an essential aspect of construction projects, where cost is seen as a crucial factor in project feasibility studies and early decision-making. Pre-project cost estimate accuracy is a critical factor in the success of any construction project, and projects often suffer from issues such as cost overrun, especially in the case of projects with a tight budget. Cost overrun can potentially result in the cancelation of projects and excessive delays.

The costs of a building project need to be estimated with high precision. However, lack of sufficient prior knowledge and dynamically changing factors having a direct impact on the overall project cost are the major barriers in the cost estimation of a cost, particularly in the early stages. As such, cost-estimating methods are used to address this lack of detail, providing results within a reasonable range of accuracy [77].

AI, especially neural networks, plays a vital role in building good-quality highways and roads at a faster pace with less labor and cost. Estimation of the cost of building highways and roads before actually starting the construction work is very crucial. Substantial time, money, and manpower are spent in doing these calculations. AI can instead be used for the automatic estimation of road construction parameters from which the building estimate cost can be calculated easily.

Cost estimation of highway projects using parameters is very beneficial in the early stages when very minimal information is known. Parametric-based project cost estimation involves identifying the key parameters along with their importance such that the parameter with the highest importance is given the highest weight. These weighted parameters are then input to an AI-based model, which tries to predict the cost of the whole project. Along with the cost and the offered price, another factor in choosing the best contractor for a construction project is the proposed time of completion. The timely completion of any project is of utmost importance and is a crucial factor of consideration, along with the offered price, when awarding the bid to a contractor.

**Figure 9:**
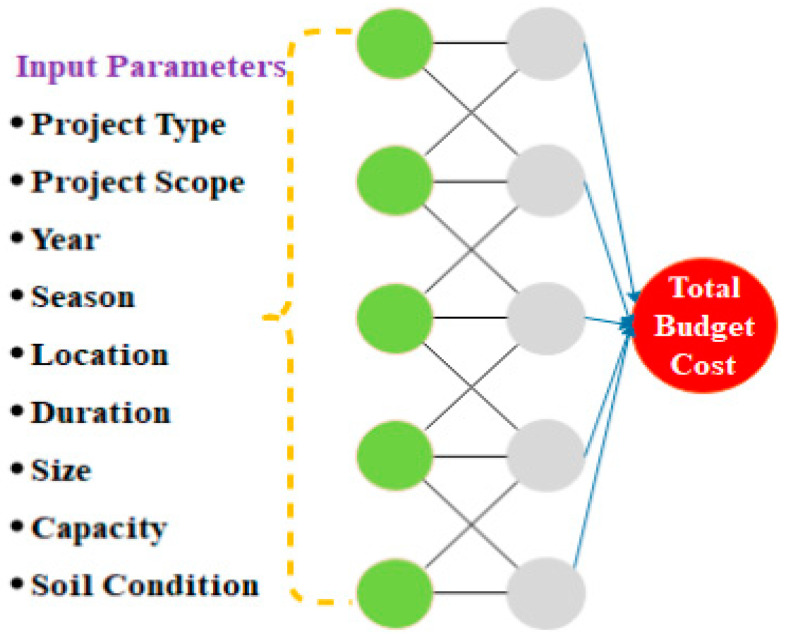
Neural network for prediction of the budget cost of a project.

The authors in [78] introduced a three-layer neural network for the automatic estimation and prediction of the cost of building a highway project. This AI-inspired model for road cost prediction is transparent and easy to use for practitioners in construction. This neural network model was implemented in Microsoft Excel in the form of a spreadsheet simulation. In their work, the spreadsheet represented a template for a neural network with one hidden later, and standard steps such as (a) data organization, (b) data scaling, (c) weight matrix, (d) output of hidden layers, (e) final output of neural network, and (f) backpropagation of errors were performed for the processing of the template. This work made use of bids of 18 different highway projects submitted in the span of 5 years in the office of Public Works, Services, and Transportation, St. John’s, Newfoundland, Canada. Each bid had the itemized cost of various jobs with the identity of bidders not disclosed. Some contractors were also contacted to gather cost-related information required for training the model. The authors identified 10 key attributes crucial for estimating the project cost using the AI-based models. These attributes were (a) descriptors of project size, (b) project type (subclassified as (i) bridge, (ii) highway, and (iii) others), (c) construction season (i.e., winter, summer, or fall), (d) location, (e) duration of project in months, (f) size of highway/road in km, (g) capacity (e.g., two lanes or two lanes divided), (h) water body (yes or no), (i) soil condition, and (j) year. The representation of the work is shown in Figure 9. In order to determine the optimal weights of the neural network, techniques such as simplex optimization, backpropagation, and genetic algorithm (GA) were used by the authors in this model. According to the their conclusion, it was found that the simplex optimization technique was able to produce the most optimal neural network for determining and predicting the cost of a national highway project.

Bidding is the primary process to decide the contractor to which a highway or road project is to be given. Thus, it is required for a contractor or a bidder to furnish an optimal bid for doing a construction-related task. In order to provide a close to accurate prediction of the project cost, the authors in [79] proposed an AI-inspired model for the prediction of the project’s cost. In this work, the authors used various data mining and AI-based algorithms such as multiple regression, GA, case-based reasoning (CBR), and ANN for the prediction of bid award amount on the basis of limited available information. For the training of the AI models, data from various bridge construction-related projects were fetched from the database of Taiwan Public Construction Commission. Initially, the authors fetched 275 bridge construction projects, which was reduced to 98 projects of interest after the application of various filters to check for projects within the scope of this work. Features such as unit price analysis tables, design drawings, construction contracts, and detailed price quotes, important for the prediction of bid award amount, were extracted from the dataset. For validation of the performance of the model, the authors used a k-fold cross-validation technique in which the dataset was divided into 10 subsets of similar sizes, whereby, after the removal of one subset, the nine remaining subsets were used for learning. The error rate was computed using the holdout set. The whole learning process was executed a total of nine times for different training sets, with each set removed. The accuracy of the algorithm was computed as the average accuracy of the 10 models in 10 validation rounds. The mean absolute percentage error (MAPE) was the metric used for evaluating the performance of the model. From the performance analysis, it was observed that the GA- and ANN-based models showed promising performance in the prediction of highway cost amount. The linear regression’s performance was not found to be optimal in the results. According to the test data, the MAPE values of GA–ANN, CBR, and linear regression were 7.526%, 8.83%, and 9.149%, respectively.

Similarly, for estimating the cost and duration of urban road construction projects, the authors in [80] proposed an AI-based prediction model using SVM and ANN. Correct prior estimation of the total funds and resources required in a project can help constructors to fix the best bid for a project, inclusive of the own profit margin. Furthermore, the prior estimate of time required for the completion of a project can help the constructor and funding organizations determine the completion time of a project. For the proposed model to predict the cost and duration of a road construction project, the authors gathered training data from various realized road construction projects carried out between January 2005 and December 2012 in the city of Novi Sad, Republic of Serbia. In total, 198 realized projects were collected, which was later reduced to 166 after ruling out projects which were not related to road construction. For estimating the cost, inputs fed to the model included the amount of crushed stone, number of curbs, amount of asphalt base and surface layer, preparation works, earthworks, drainage works, and traffic signalization works. The complete model for estimating cost and duration was simulated using the Statistica 12 software package. From the simulation results, it was observed that, due to the different impact of input parameters on the cost estimation in contrast to the project’s duration, a greater accuracy level was achieved when using separate models for their estimation. A combined model resulted in lower precision in the case of ANN, whereas SVM was able to provide better generalization and greater accuracy in the estimation of both cost and duration.

Another study predicting the duration and cost of highway road projects was proposed by the authors in [81] using ANN. Data for model training were taken from past projects in which all items were uniform. The proposed ANN-based predictive cost and duration model considered bills of the following items for prediction: earthwork, site clearance, sub-base works, culverts, bituminous works, minor and major bridges, junctions and curbs, drainage works, traffic signs, vehicular underpass (VUPs), pedestrians underpass (PUPs), and return walls, miscellaneous items, robs, flyovers, and overpasses, toll plazas, and street lighting in urban areas. For evaluation of the performance of the ANN-based model, the authors compared the predicted value for each input item with its corresponding actual cost and duration. The ANN architecture consisted of a first layer with 10 neurons and trainlm as the training function, followed by 10 hidden layers and one output layer. The whole implementation of the ANN-based model was done using the MATLAB R2013a software. According to the simulation results, it was observed that the model gave MAPE scores of 0.57% and 0.27% for cost and duration, respectively; thus, it was found to be ideal for use by construction companies for the accurate prediction of project cost and duration with less information.

The authors in [82] stressed upon the importance of correctly predicting and estimating the cost of a project in its early stages to help in winning bids and making perfect bids while considering their profits and margins. To do this, the authors used ANN on a dataset featuring 132 completed engineering service-related projects with 16 different inputs which were assigned different weights according to their importance. Some of the input factors considered for cost prediction by the authors are shown in Table 3. MAPE was used for evaluating the performance of the ANN-based cost predictive model, and, from the simulation results, it was clearly observed that ANN-based models can easily be applied to produce accurate predictions even with little input.

Other research work available in the literature for the prediction of cost and duration of a construction-related projects using AI techniques is mentioned in Table 4.

## 5. Important Input Parameters, AI Techniques, and Performance Metrics for the Estimation of Cost, Time, and Shear Strength

As seen in previous sections, the great success obtained when using different types of ANN-based models in various construction-related applications such as early prediction of project cost and duration and estimation of soil shear strength is quite evident. In all these applications of AI models for the accurate prediction and estimation of crucial construction-related parameters, certain inputs with a huge impact on the prediction accuracy are required.

The influential factors of cost are elements that reflect a building’s features and impact its cost. A project is characterized by influential factors, and it is possible to characterize a project more accurately as the number of influential factors increases. Nevertheless, if the number of significant variables is high, the number of cases for estimation is also high. Furthermore, increasing the number of input variables for cost prediction will not necessarily increase the accuracy of the calculation. Therefore, it is necessary to select the correct number of factors to enhance the effectiveness of estimation.

In this section, the limited information that can be used for construction-related predictions is discussed. The variables were identified and defined through an intensive literature review and expert interaction for input into some ANN-based models. Different applications require specific input information for prediction. For instance, for computing the expected duration of a project, crucial parameters such as the project size, labor size, type of project, and terrain type are usually considered. Similarly, for computing soil shear strength, inputs such as clay percentage are taken into consideration.

For the selection of these inputs, various construction engineers and contractors were consulted, and variables identified through a literature review with a higher frequency of usage and availability at the early stage were considered for selection.

Additionally, there are other factors that can help in improving the accuracy of these early cost and duration prediction models, as listed below [86,87,88,89,90,91,92].

1. *Project complexity:* A project whose cost and duration is to be estimated must be analyzed properly to check for all potential cost-incurring activities. The complexity of a project can be determined in terms of the number of repetitive tasks, its size, the kind of work, the number of operations involved, etc. The project’s complexity has an impact on its duration and eventually on the overall construction cost. A more complex project requires more time and, thus, incurs a greater cost. Similarly, the size of a project also has a great impact on its cost. If the size of a project (calculated in terms of square feet or meters) increases, the amount of labor that needs to be employed to get the work done also increases, which eventually adds to the cost of the project [65,93,94].
2. *Clear specification:* A clear and detailed specification can prevent any information from being missed, thus improving the cost and duration prediction accuracy.
3. *Prior experience of the contractor and staff for cost estimation:* An experienced contractor and an experienced team are very crucial for cost estimation.
4. *Equipment requirements:* The choice of equipment is very crucial for governing the cost and duration of the project. Any later change in the list of equipment due to factors such as unavailability or poor performance can affect the overall cost and duration of the project.
5. *Clear scope definition:* A clear definition of scope is critical to focus on the client’s specifications and requirements. This allows deciding the correct project team to manage the expense and length of the project. The project team and estimators should, therefore, remove any uncertainty in the scope and make it transparent and understandable.
6. *Consideration of site constraints:* The estimator should consider different site constraints such as access to resources, storage, and services as these could incur extra charges in certain scenarios in comparison to the original cost estimate. The site is critical to the project; thus, its constraints should be fully analyzed for cost elements which are unique with the greatest impact on the estimate of costs.
7. *Availability of material:* When making the cost and duration estimate, the estimator should check for the availability of material to be used in the project. Unavailability of material during the project can force the contractor to purchase from another supplier, which can add to the project costs that were not included in the initial estimate, while also impacting the duration of the project.
8. *Availability of and consultation with previous similar bids:* The estimator should consider previous similar bids and should try to identify the necessary activities along with their prices before making the cost estimation of a project.
9. *Change in currency exchange rate:* Fluctuations in the currency exchange rate can sometimes affect the cost of the project and lead to the issue of cost overrun.
10. *Number of competitors:* According to various studies [91,95,96,97,98,99], it was observed that increases in the level of competition lead to excessive cost overrun. The number of competitors can be computed as the total number of bidders who file their bid for a project. Bidders often quote unrealistic values for a project in order to achieve the lowest bid for the project, leading to cost overrun during the project at later stages.
11. *Type of client:* Since each construction project has its own ideas, tasks, and goals in accordance with the client, the specifications of every contract and the bidding behavior are majorly influenced by the type of client. There exist different types of client such as the government, large developers, medium/small-scale retailing organizations, large-scale commercial organizations, and other public- and private-sector clients [65,100].
12. *Financial status of the contractor and owner:* Construction work requires high daily expenses, and, when payments are overdue, most contractors are unable to meet these expenses. Due to delays in payments by the client, work progress can be slowed owing to insufficient cash flow to cover the contractor’s construction expenses. This problem is particularly serious for contractors who are not economically viable [96,101,102].
13. *Frequent changes in design specification:* Frequent changes in the design specification of the project as per the demand of the client and/or designer usually adds new modules during construction, leading to wastage of time and material and subsequent cost overrun.
14. *Material costs and their fluctuation:* Correct and prior selection of material in terms of its cost has a huge impact on the cost of the project; thus, the choice of the material should be made wisely considering the cost, availability, ease of use, and performance factors. Thus, any optimal method employed for material selection will reduce wastage and improve the project cost. Similarly, fluctuation in prices after bid approval can lead to cost overrun of the project. This may be due to monopoly, supply and demand, inflation, and political scenario [96,103,104].
15. *Awarding the contract to the lowest bidder:* Owners generally grant project contracts to the lowest bidders; however, these are typically poorly skilled contractors who are tight on funds. This leads to poor results and delays in completing the job, thereby increasing the overall cost and duration of the project. Pre-qualification criteria and policies followed when granting the project need to be strengthened to prevent this problem. This can also lead to cost overrun [96,105].

### 5.1. Crucial Parameters Affecting Estimation of Soil Shear Strength in a Construction Project

1. ***Clay content:*** Clay is an important geotechnical engineering material and comes under the category of fine-grained soil. Clay generally has numerous issues such as a high level of volumetric changes, high compressibility, and low strength. Thus, clay needs to be improved before actually using it for the construction of roads, dams, waste landfills, and slurry walls, etc. Enhanced gradation, reduction in plasticity and swelling capacity, and increased strength and workability typically enhance clay stability [106]. The content of clay in the soil affects its plasticity and, thus, reduces its shear strength [71,107]. Clay content can be mathematically calculated as follows [108]:
  (1) $$ClayContent=\frac{m_{0.005}}{m},$$
  where m is the mass of the soil sample, and $m_{0.005}$ is the mass of soil particles falling through the 0.005 mm sieve.
2. ***Plastic limit:*** The plastic limit also has an impact on the soil shear strength. It is defined as the percentage of water and the moisture content at which the soil starts to crumble and change from a semisolid state to a plastic state [109]. An increase in plastic limit causes a decrease in soil shear strength [71,107]. The plastic limit can be mathematically calculated as follows:
  (2) $$PlasticLimit\%=\frac{mass_{plastic}}{mass\;of\;particles\;in\;sample}\times 100,$$
  where $mass_{plastic}$ is the mass of water content in the sample at which the soil starts to change from a solid to plastic state.
3. ***Moisture content:*** The moisture content of soil can be defined as the ratio of the amount of water held in the soil to that in dry soil [110]. The mass of water can be computed as the difference before and after drying the soil. Moisture content has an impact on the soil shear strength, whereby a greater moisture content leads to lower cohesion between the soil particles and, thus, a weaker soil.
  (3) $$MoistureContent\%=\frac{mass\;of\;water\;in\;soil}{mass\;of\;particles\;in\;sample}\times 100.$$
4. ***Specific gravity:*** The specific gravity of a soil is defined as the ratio of its particle density to the density of its water content. Soils with a higher specific gravity have a high shear strength as heavy particles are present in the soil, thus leading to compact and strong structures [111]. This parameter can be calculated using the following mathematical equation:
  (4) $$SpecificGravity=\frac{density\;of\;soil\;particles}{density\;of\;water}.$$
5. ***Liquid limit:*** The liquid limit is defined as the moisture level at which the soil’s state begins to change from plastic to liquid [112]. The soil shear strength decreases with an increase in the liquid limit [71,107]. It can be calculated using the following mathematical equation:
  (5) $$Liquid\;Limit=\frac{m_{liquid}}{mass\;of\;soil\;particles}\times 100,$$
  where $m_{liquid}$ is the mass of water in the soil at which its state begins to change from plastic to liquid.
6. ***Silt percentage:*** Silt is medium in size and has a smooth texture. This parameter refers to the amount of silt in the considered soil sample.
7. ***Sand percentage:*** Sand, silt, and clay are types of soil, and the difference lies in their size. Sand is the largest type and feels gritty, whereas silt is of medium size with a smooth texture, and clay has the smallest particles and is sticky in nature [113]. Sand percentage refers to the sand content in the sample soil.
8. ***Plastic index:*** The plastic index is an indicator of the soil’s plasticity, defined as the water content at which the soil shows plastic properties. The plastic index is calculated as the difference between the liquid limit and the plastic limit. Soil having a high plastic index tends to be clay, while soil with a low plastic index tends to be silt. A plastic index of 0 indicates the presence of very little or no silt or clay. Thus, the plastic index basically allows determining the type of soil and the degree of cohesion it exhibits.
  (6) Plastic Index=Liquid Limit−Plastic Limit.
9. ***Liquid index:*** This is the ratio of the difference between a given soil’s natural moisture content and the plastic limit to the difference between the liquid limit and the plastic limit.
  (7) $$Liquid\;Index=\;\frac{Soil’s\;Moisture\;Content-\;Plastic\;Limit}{Liquid\;Limit-Plastic\;Limit}.$$
10. ***Dry density:*** Dry soil density reflects the ratio of total dry soil mass to total soil volume. Dry density is correlated with the degree of compaction of the soil surface. A high degree of compaction denotes a high dry density of the soil.
  (8) Dry Density=Wet Density− Moisture Content.

**Figure 10:**
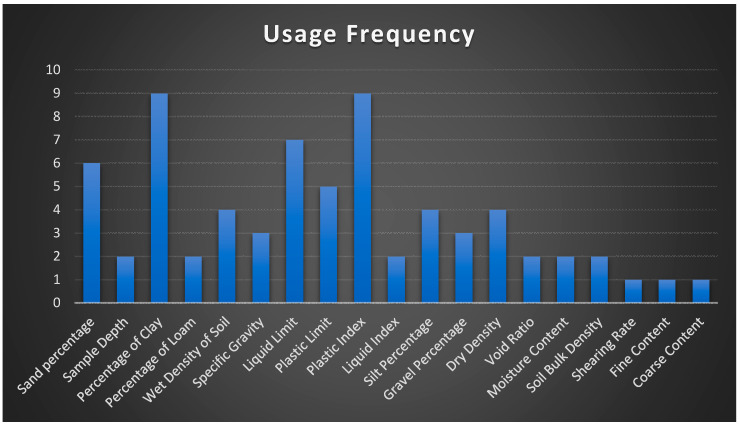
Usage frequency of input parameters for soil shear strength.

Figure 10 and Table 5 list various commonly used input parameters for the estimation of soil shear strength, along with their usage frequency in the existing literature. It can be seen that input parameters such as the percentage of clay, plastic index, liquid limit, percentage of sand in soil, silt percentage, and dry density are the most frequently used and crucial parameters in deciding the soil shear strength.

### 5.2. Crucial Input Parameters Affecting Cost Prediction in a Construction Project

By consulting engineers and experts, the authors in [114] came up with 12 sets of input cost variables, which are believed to be crucial in the preliminary cost prediction of a highway construction project, especially in the case of the Ethiopian highway construction industry. These variables are listed below.

- Project type;
- Project complexity;
- Project location;
- Project scope;
- Project size;
- Site topology;
- Bridge type;
- No. of bridges;
- Existence of ground water;
- Soil type;
- Inflation rate;
- Project duration.

**Figure 11:**
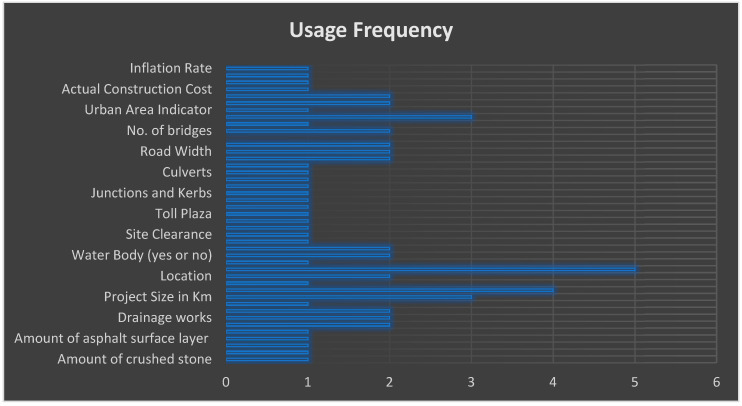
Usage frequency of input parameters for construction cost prediction.

An exhaustive list of input parameters for project cost prediction is given in Table 6, along with their frequency of usage in the literature. A graphical depiction is shown in Figure 11. According to the analysis, it was observed that parameters such as project duration, type of project (e.g., bridge, highway, or other), size, and geographical location are the input parameters widely used by researchers for the prediction of project cost.

### 5.3. Crucial Input Parameters Affecting Cost Prediction in Maintenance of a Building

The input parameters that can be used for the prediction of costs involved in building maintenance can be categorized as initial input costs and base costs. A list of useful parameters for deciding the maintenance cost of an existing building is shown in Table 7. Various parameters contributing to the initial input costs are as follows:

1. ***No. of floors:*** This is the total number of floors of a building in a construction site that requires maintenance. A greater number of floors leads to a higher maintenance cost.
2. ***Floor height:*** The floor height is another crucial parameter for computing the cost required for project maintenance.
3. ***Total building area:*** This is another important parameter with a huge impact on deciding the maintenance and construction cost of a building. It is calculated as the sum of the floor area of all floors in all buildings on a site.
4. ***Year of build:*** The year the structure was constructed (i.e., how old the constructed building or project is) can be used to determine the level of maintenance required for that structure. Older buildings or structures require additional effort, labor, material, and cost in comparison to newly constructed projects.
5. ***Structure type:*** The structure type can be categorized as steel-framed wall or concrete-bearing wall.
6. ***Envelope type:*** The building envelope is a complicated yet integral entity and comprises all the exterior components of a building, including its roof, walls, below-grade waterproofing, windows, and skylights. The building envelope must be correctly engineered, constructed, and maintained to avoid the absorption of water and air through the envelope and to restrict condensation.

Parameters categorized as base costs include equipment costs, contractor fees, architecture fees, and furnishing costs. They can vary from one geographical location to another. The initial cost and the base cost define the total cost required for a maintenance-related project.

Table 7 lists the commonly used input parameters when deciding the maintenance cost of a building.

### 5.4. Discussion

**Figure 12:**
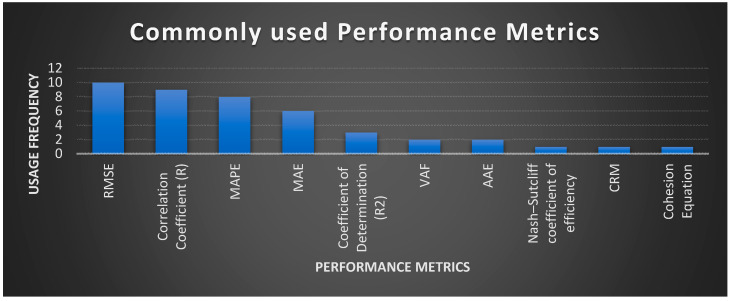
Commonly used performance metrics in the prediction of soil shear strength and project cost.

According to the analysis drawn from the literature review with a focus on the prediction of soil shear strength and pre-project cost and duration using AI techniques, it was observed that most authors used performance metrics such as RMSE, correlation coefficient, MAPE, and MAE for validating the efficiency of their proposed AI-based prediction models. A complete list of performance metrics along with their usage frequency is shown in Table 8 and Figure 12.

**Figure 13:**
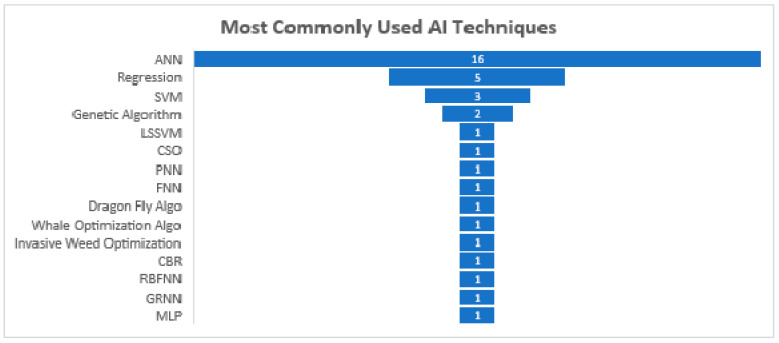
Most commonly used AI techniques in the prediction of soil shear strength and project cost and duration.

Furthermore, according to the usage frequency of various AI-based models, it was found that ANN topped the list for the prediction of soil shear strength and pre-project cost and duration, followed by regression- and SVM-based models. A complete list of AI-based models used by various articles on the basis of their usage frequency is shown in Table 9 and Figure 13.

## 6. Challenges in the Use of AI in Construction-Related Activities

In this section, various challenges that can act as barriers in the performance of AI for the estimation of soil shear strength and initial project cost, along with the limitations found in existing work, are presented. These challenges need to be considered and addressed for improving the overall performance of prediction systems in geoscience and construction applications.

- *Availability of the same parameters in all projects:* The parametric cost prediction of projects in the early stages has some serious issues and challenges which require consideration. The first challenge is the nonavailability and applicability of the same cost estimation parameters in all projects. Some parameters which are marked as crucial in one project for the cost modeling and training of the prediction framework may not be applicable or available in other projects. Furthermore, these parameters may vary with geographical area. Thus, the AI model is expected to adapt on its own through a sensitivity analysis to generate the most accurate cost with fewest prediction errors.
- *Not much work on prediction of project duration:* According to the literature review of AI applications in construction-related projects, it was observed that not much work has been done on the prediction and estimation of construction project duration, in contrast to cost estimation. Moreover, most existing databases used by researchers for estimating project duration failed to break the project down into specific work activities, instead presenting the duration estimate of the whole project.
- *Sensitivity analysis of the model:* It is well known that, for an AI-based prediction model to work, certain input parameters with a huge impact on the prediction need to be included. However, certain crucial input parameters may not be present; thus, the model should be made sensitive enough such that the absence of certain input parameters does not affect its prediction result.
- *Standard validation methods:* There should be a standard validation method for evaluating the performance and accuracy of cost estimation and soil shear strength calculations. No uniformity was observed in the choice of performance metrics by researchers in the literature. As such, standard validation metrics for measuring the performance of AI-based prediction models should be developed such that their performance can be computed at a similar level.
- *Standard input parameters for estimation:* According to the survey conducted, it was observed that different input parameters were used by different researchers in their work for shear strength estimation and pre-construction project cost estimation. It is well known that very limited prior information is available during the estimation of these factors. However, there should be a list of some standard input parameters that are believed to be crucial for the prediction of the abovementioned applications, along with their importance, allowing potential replacements for unavailable parameters.
- *Lack of proper scientific justification:* It was observed that, in some studies, there was a lack of proper scientific justification for the results obtained after the application of AI models. Moreover, details on features with the greatest and lowest contribution to the result were found to be missing.
- *Handling of missing data:* Missing data were often not properly managed, thus necessitating clean databases.
- *Small datasets for model training:* It is well known that AI models are trained using existing databases. For an AI-based model to make correct predictions, there must be ahigh-quality dataset covering all possible cases of the problem for which it was trained. However, it was observed that, in most studies, the size of the database was not adequate.
- *Cost and duration overrun:* Cost overrun is one of the major challenges faced by construction projects due to the various factors mentioned in Section 5. AI-based prediction models can be efficient for pre-project cost and duration estimations; however, highly dynamic factors such as geolocation, climatic changes, and natural disasters can hugely affect these prediction models. Thus, it is very much required to train the AI models using datasets from that specific location only.
- *Issues of AI-based models:* AI models suffer from various issues such as overfitting, underfitting, hyperparameter selection, and optimization issues. Therefore, in order to deal with such issues and obtain the most accurate results, multiple cost prediction models using different AI techniques are required to be developed and considered, such that the prediction results of each are compared to obtain the best model with the most accurate result for the chosen scenario.
- *Factors affecting the construction project cost and duration differ from location to location:* It is well known that parameters for project cost and duration estimation differ from location to location, e.g., the cost of labor and materials. Thus, a model trained with a dataset of past projects from a country such as Norway may lead to cost overrun and misquotation in another country such as the United Arab Emirates. Thus, such challenges need to be addressed.

## 7. Conclusions

In this study, an attempt was made to highlight the capability of AI in the field of geotechnical and civil engineering. AI has already revolutionized our day-to-day life, and its applicability can easily be seen in various important sectors such as healthcare, robotics, defense, intelligent transportation systems (ITS), and agriculture, due to its ability to solve complex problems with great performance and efficiency. AI can also be used for making accurate and quick predictions of cost and duration in various construction-related applications through the use of various inputs. This prior information is usually limited in nature; however, pre-project cost and duration estimations play a very important role for both bidders and clients in construction tasks. This can greatly help in making the best and most genuine bid for a project after including the profit margin, preventing the common issue of cost overrun. Similarly, the estimation of soil shear strength before construction is very crucial for determining its capability to withstand high external forces such as floods and earthquakes. Accordingly, in this article, the focus was laid on the use of various powerful AI models for the estimation and prediction of these two important applications of geotechnical engineering.

Various existing research articles related to the use of AI for the prediction of soil shear strength and pre-project cost and duration were studied, and various input pre-project parameters were enumerated. According to the survey conducted, it was found that, among the various AI models and techniques, ANN was the most used model followed by regression and SVM. This is due to ANN’s ability to provide a generalized optimal solution with high accuracy and in less time; they have huge support and can be implemented easily. In the performance evaluation of various AI models in the prediction of soil shear strength and pre-project cost and duration, metrics such as RMSE, correlation coefficient (*R*), MAPE, MAE, coefficient of determination (*R^2^*), VAF, and AAE were the most used metrics.

It is well known that the knowledge of pre-project parameters is limited; however, they play a very crucial role in the accurate estimation of the abovementioned applications. Thus, according to the literature review, it was found that, for the estimation of soil shear strength, input parameters such as the percentage of clay, plastic index, liquid limit, sand percentage, plastic limit, wet density of the soil, silt percentage, and dry density are crucial. On the other hand, the project duration, project type (bridge, highway, or others), project size, location, geographical complexity, road length and width, soil condition, etc. are crucial input parameters greatly affecting the project cost.

Various factors, both internal and external, that can lead to issues such as cost overrun, were also listed in this article. A sensitivity analysis was performed by several authors to see how an AI model would perform if one or more input parameters were not available. An AI model should be robust and adaptive enough to work without greatly affecting its performance, even in the absence of some input parameters.

Lastly, various challenges and issues facing the prediction of these geotechnical applications, which can affect the performance of these AI-based models, were presented. This article can serve to motivate and assist researchers and geotechnical engineers using powerful AI technology in various applications of civil and geotechnical engineering. Future aspects of this study can be to explore more applications of AI in civil and geotechnical engineering, as well as in construction applications, to enumerate real-time challenges.

## Figures and Tables

**Table 1:** Comparison of artificial intelligence (AI)-assisted vs. manual construction of roads and buildings.

Parameters	Artificial-Intelligence-Assisted Road and Building Construction	Manual Road and Building Construction
Labor	Less labor is required as the manual and repetitive work will be automated by the machine.	Labor is required for the work to be done, which incurs both time and cost.
Cost	Cost is reduced as labor is replaced by automated machines. Moreover, automated steps reduce the need for the use of equipment required in the manual construction process. AI also reduces the material and time wastage with its accurate calculations and, thus, reduces the overall cost of the production.	Cost is required for paying the bills related to extra labor, equipment, cost due to delay in project, material wastage, etc.
Time	AI ensures the timely completion of the project through the prior prediction of the project duration. Most time-consuming tasks are replaced by AI-assisted technology, which speeds up the overall project activities.	The manual construction process is time-consuming as it is greatly dependent on human labor, which is prone to factors such as the unavailability of skilled labor and errors.
Accuracy	AI is highly accurate and uniform in its predictions and estimations as it takes into account various input parameters and factors that affect the predictions and estimations of an output.	Accuracy depends upon the experience and skill of the person making the predictions and estimations. Moreover, it is not possible to manually consider all input parameters as it is too complex a process.
Risk	There is low risk to human lives as the repetitive tasks, as well as tasks which humans are reluctant to perform where their lives are at risk, are performed by the machine.	There is high risk involved in dangerous construction-related jobs.
Cost-Overrun Issues	There are no cost-overrun issues as AI-based models make very accurate predictions of the project cost and duration by considering various crucial input parameters with impact on the overall cost of the project. Furthermore, sensitivity analysis is also performed, which improves the robustness and accuracy of the prediction model when certain input parameters are available.	There are often cost- and duration-overrun problems because of issues such as the lack of experienced cost estimators and the inability to consider all crucial parameters.

**Table 2:** Artificial intelligence for the estimation of soil shear strength.

Ref.	Application	Artificial Intelligence Techniques Used	Parameter Computed	Dataset Used	Input Parameters	Performance Metrics	Simulation Software Used	Performance Results
[66]	Predicting soil shear strength for road construction	Hybrid AI using LSSVM and CSO	Soil shear strength	332 soil samples collected from Trung Luong National Expressway Project, Vietnam	Sand percentage Sample depth Percentage of clay and loam Wet density of soil Specific gravity Liquid limit Plastic limit Plastic index Liquid index	RMSE MAPE VAF Coefficient of determination (*R^2^*)	MATLAB along with the LS-SVMLAB toolbox	RMSE: 0.078 MAPE: 14.841% VAF: 93.110% *R^2^*: 0.885
[67]	Predicting soil shear strength for road construction	ANN and regression tree	Soil shear strength (cohesion and internal friction angle)	115 soil samples with 95 soil samples for training while 20 for testing	Sand percentage Silt percentage Gravel percentage Clay percentage Plastic index Dry density	Correlation coefficient (*R*) RMSE	MATLAB	ANN *R*: 0.87 RMSE: 0.136 Regression tree *R*: 0.73 RMSE: 0.162
[64]	Predicting soil shear strength for road construction	Probabilistic neural network (PNN)	Soil shear strength (cohesion and internal friction angle)	300 soil samples from different 20 bore holes in Ranchi, Jharkhand, India	Soil water content Plasticity index Dry density Gravel percentage Sand percentage Silt percentage Clay percentage	Observed cohesion and predicted cohesion Observed friction angle and predicted friction angle	Not mentioned	The difference between the predicted and observed cohesion and predicted angle was between 7% and 14%.
[68]	Predicting soil shear strength for road construction	Functional neural network (FNN)	Residual strength of clay	131 samples of database obtained from areas of landslide, debris flow, volcanic eruptions	Liquid limit, Plasticity index Clay fraction	Correlation coefficient Nash–Sutcliffe coefficient of efficiency Absolute average error (AAE) Maximum average error (MAE) Root-mean-square error (RMSE)	MATLAB	Best case while using all the input parameters, *R*: 0.898 RMSE: 2.782
[69]	Predicting soil shear strength for road construction	ANN and SVM	Residual friction angle of clay	Database obtained from areas of landslide, debris flow, volcanic eruptions	Clay fraction Liquid limit Plasticity index	Correlation coefficient RMSE MAE AAE	MATLAB	RMSE: 7.0
[71]	Predicting soil shear strength	SVM	Soil shear strength	538 samples of soil collected from Long Phu 1 Power Plant Project, Soc Trang Province, Vietnam	Clay content Moisture content Specific gravity Void ratio Liquid limit Plastic limit	Correlation coefficient RMSE MAE	MATLAB using the machine-learning toolbox	SVM performed well for the prediction of soil shear strength with a correlation coefficient between 0.9 and 0.95. Moisture content, liquid limit, and plastic limit were found to be the most important parameters.
[72]	Predicting soil shear strength	ANN	Soil shear strength parameter of friction angle	320 samples obtained from Geotechnical Engineering laboratory of the Federal University of Bahia (UFBA), Brazil	Sand content Plastic limit Coarse content Fine content Liquid limit Soil bulk density Shearing rate	Coefficient of determination (CoD) RMSE Coefficient of residual mass (CRM)	Not mentioned	Sensitivity analysis was also performed to check how the system would respond if certain input information was not available. Soil bulk density was found to be an important parameter. RMSE: 51.63 CRM: 0.00518 CoD: 0.97
[73]	Predicting soil shear strength	Three nature-inspired hybrid algorithms, i.e., dragonfly algorithm, whale optimization algorithm, and invasive weed optimization of ANN	Soil shear strength	28 boreholes were constructed and 154 soil samples were obtained from Royal City Project of Hanoi, Vietnam	Depth of sample % of sand % of loam % of clay % of moisture content Wet density Dry density Void ratio Liquid limit, plastic limit Plastic index Liquidity index	RMSE MAE CoD	MATLAB 2014	*RMSE* ANN: 1 DFA: 4 WOA: 2 IWO: 3 *MAE:* ANN: 1 DFA: 2 WOA: 3 IWO: 4 *CoD:* ANN: 1 DFA: 4 WOA: 2 IWO: 3
[74]	Predicting soil shear strength	ANN	Soil shear strength (cohesion and internal friction angle)	83 soil samples were collected from random locations of central and southern areas of Delta State	Plasticity index percentage Liquid limit Specific gravity	MAE RMSE Coefficient of correlation Cohesion equation	Visual Basic software	RMSE: 8.33 MAE: 6.08 Coefficient of correlation: 0.861
[75]	Predicting soil shear strength	ANN	Soil shear strength (cohesion and internal friction angle)	20 boreholes were detected and 200 soil samples were collected from Nalanda District of Bihar, India	Plasticity index Sand percentage Silt percentage Clay percentage Bulk density Dry density Water content	RMSE Correlation coefficient (*R*)	Not mentioned	RMSE: 0.636 MAPE: *R*: 0.907
[76]	Predicting soil shear strength	Multivariate regression and ANN	Soil shear strength (cohesion and internal friction angle)	108 soil samples taken from Isfahan, Iran	Plastic limit Liquid limit Plasticity index Density Clay % Silt % Sand % Gravel %	RMSE MAE Variance accounted for (VAF)	SPSS 23 software	Correlation coefficient analysis showed that liquid limit, plastic limit, and % of clay and silt were important input parameters for the calculation of soil cohesion, whereas density, plasticity index, liquid limit, and % of clay, sand, and silt were crucial for the prediction of effective friction angle.

**Table 3:** Some input parameters for cost prediction.

Input Parameter	Description	
Scale of work	Actual cost of construction	
Project phases	Master plan Basic design/detailed	Conceptual design Detailed design
Project duration	No. of days the project will take	
Scope of work	List of activities included in the contract	
Type of work	Modification/maintenance or new construction	
Client’s expertise	The level of experience on client’s side	
Size of project team	No. of team members	
Multidisciplinary nature	No. of disciplines involved	
Type of client	How demanding client is for standard	
Main market type	Oil and gas Chemicals Energy and environment	Infrastructure Industrial property Public sector
Attitude toward design changes	Cooperative or noncooperative	
Project manager’s experience	The number of hours of experience	
Contract type	Fixed price or reimbursable	
Intensity	Average hours that the team members work	

**Table 4:** Artificial intelligence for project cost estimation.

Ref.	Application	AI Techniques Used	Construction Parameter Computed	Dataset Used	Input Parameters	Performance Metrics	Simulation Software Used	Performance Results
[78]	Prediction of road building cost	Neural network and genetic algorithms	Budget cost estimate of a project	Bids of 18 different national highway projects submitted over the span of 5 years in the office of Public Works, Services and Transportation, St. John’s, Newfoundland, Canada	Descriptors of project size Project type subclassified as (i) bridge, (ii) highway, and (iii) others Construction season, i.e., winter, summer, or fall Location Duration of project in months Size of highway/road in km Capacity, e.g., 2 lanes or 2 lanes divided, Water body (yes or no), Soil condition	Error in cost estimation Prediction error Sensitivity analysis	Microsoft Excel	Weighted errors Backpropagation: 10.4% Simplex optimization: 1.0% Genetic algorithm: 21.8%
[79]	Prediction of road building cost and duration	Genetic algorithm (GA), multiple regression, artificial neural network (ANN), and case-based Reasoning (CBR)	Budget cost estimate of a project	Bid invitation and bid award data of 98 bridge construction projects obtained from Taiwan Public Const. Commission database from June 2008 to May 2009	Design drawings Unit price analysis tables Detailed price quotes Construction contracts	Mean absolute percentage error (MAPE)	ANN using Decision Tools Software Evolver Software for GA modeling	MAPE values GA–ANN: 7.526% CBR: 8.83% Linear regression: 9.149%
[80]	Prediction of road building cost and duration	ANN and SVM	Project cost and duration estimate	166 completed construction related projects carried out between January 2005 and December 2012 in Novi Sad, Republic of Serbia	Amount of crushed stone Number of curbs Amount of asphalt base layer Amount of asphalt surface layer Amount of concrete prefabricated elements Percentage share of work positions Preparation works Earthworks Drainage works Traffic signalization works Other works Work realization zone Project category (values of up to and over 40,000,000)	MAPE	Statistica 12 software package	SVM outperformed the ANN-based model in calculation of estimate cost in terms of MAPE with values of 7.06% and 25.38%, respectively. For estimate of project duration, which proved to be a challenge, SVM and ANN produced MAPEs of 22.77% and 26.26%, respectively.
[81]	Prediction of road building cost and duration	ANN	Project cost and duration estimate	2 completed projects having the same set of resources	Site clearance Earthwork Sub-base works Bituminous works Culverts Major and minor bridges Drainage works Junctions and curbs Traffic signs Miscellaneous items VUPs, PUPs, and return walls Flyovers, robs, and overpasses Toll plaza Street lighting in urban areas	MAPE Sensitivity analysis	MATLAB R2013a software	Average MAPE values for total cost and construction period were 0.57% and 0.27%, respectively.
[82]	Prediction of road building cost and duration	ANN	Engineering service-related cost prediction	132 projects	Scale of work Project duration Type of work Level of experience on client’s side Size of project team Multidisciplinary nature Type of client and requirements Project manager experience Intensity Pre-contract design Main market type Contract type	Correlation coefficient MAPE	Not mentioned	ANN can be used for accurate cost prediction even with little available input.
[59]	Prediction of road building cost	ANN models, i.e., MLP, GRNN, and RBFNN	Project cost and duration estimate	Database of roads projects constructed in the region of Republic of Croatia	Project scope Project type Road length Road width Planned construction duration (days) Planned construction cost Actual construction cost	MAPE Coefficient of correlation	DTREG	GRNN gave the best accuracy with MAPE = 13% and coefficient of correlation = 0.9595.
[83]	Prediction of road building cost	Multiple regression analysis (MRA) and ANN	Project cost estimate	966 projects of Montana Dept. of Transportation awarded between 2006 and 2015	Roadway area Length in km Width in meters Urban area indicator Bridge type Expected contract time in months No. of bridges Geographical complexity	MAPE	NeuralTools	Top-down models offer a way to boost the predictive accuracy of cost estimate for projects having higher complexity levels and smaller sample sizes.
[84]	Prediction of road building cost	ANN	Project cost and duration estimate	1022 data from 51 highway projects of Thailand between 2002 and 2007	Traffic volume Topography Weather conditions Contract duration Construction budget % of planned and expected completion Work starting and evaluation date	MAPE	Not mentioned	Results showed that the ANN gave accurate predictions in terms of MAPE in comparison to learned methods.
[85]	Prediction of road building cost	ANN	Project cost estimate	Expressway contract data of Iraq collected between 2010 to 2014	Length in km No. of stream crossings Capacity (no. of standard width lines) No. of expressway interchanges Pavement material (flexible or rigid)	Coefficient of correlation MAPE	Neuframe	Coefficient of correlation: 90%Average accuracy %: 89

**Table 5:** List of commonly used input parameters for deciding the strength of a clay/soil.

Input Parameter	Usage Frequency	Reference
Percentage of clay	9	[64,66,67,68,69,71,73,75,76]
Plastic index	9	[64,66,67,68,69,73,74,75,76]
Liquid limit	7	[64,66,68,69,72,74,76]
Sand percentage	6	[66,67,72,73,75,76]
Plastic limit	5	[66,71,72,73,76]
Wet density of soil	4	[64,66,73,75]
Silt percentage	4	[64,67,75,76]
Dry density	4	[64,67,73,75]
Specific gravity	3	[66,71,74]
Gravel percentage	3	[64,67,76]
Sample depth	2	[66,73]
Percentage of loam	2	[66,73]
Liquid index	2	[66,73]
Void ratio	2	[71,73]
Moisture content	2	[71,73]
Soil bulk density	2	[72,75]
Shearing rate	1	[72]
Fine content	1	[72]
Coarse content	1	[72]

**Table 6:** List of input parameters deciding the construction cost of a road project.

Input Parameter	Usage Frequency	Reference
Duration of project	5	[59,78,83,84,114]
Project type subclassified as (i) bridge, (ii) highway, and (iii) others	4	[59,78,80,114]
Project size in km	3	[78,83,114]
Geographical complexity	3	[83,84,114]
Earthworks	2	[80,81]
Drainage works	2	[80,81]
Traffic signalization works	2	[80,81]
Location	2	[78,114]
Water body (yes or no)	2	[78,114]
Soil condition	2	[78,114]
Road length	2	[59,83]
Road width	2	[59,83]
Bridge type (concrete, steel, or pre-stressed concrete)	2	[83,114]
No. of bridges	2	[83,114]
Project scope	2	[59,114]
Planned construction cost	2	[59,84]
Amount of crushed stone	1	[80]
Number of curbs	1	[80]
Amount of asphalt base layer	1	[80]
Amount of asphalt surface layer	1	[80]
Preparation works	1	[80]
Work realization zone	1	[80]
Construction season, i.e., winter, summer, or fall	1	[78]
Capacity (e.g., 2 lanes or 2 lanes divided)	1	[78]
Year	1	[78]
Site clearance	1	[81]
Sub-base works	1	[81]
Street lighting in urban areas	1	[81]
Toll plaza	1	[81]
Flyovers, robs, and overpasses	1	[81]
VUPs, PUPs, and return walls	1	[81]
Junctions and curbs	1	[81]
Major and minor bridges	1	[81]
Bituminous works	1	[81]
Culverts	1	[81]
Weather condition	1	[84]
Urban area indicator	1	[83]
Actual construction cost	1	[59]
Traffic volume	1	[84]
Site topology	1	[114]
Inflation rate	1	[114]

**Table 7:** List of input parameters deciding the maintenance cost of a building.

Input Parameter	Reference
No. of floors (ground and underground)	[115,116]
Floor height	[115]
Total building area	[115,116]
Year of built	[115]
Structure type	[115,116]
Envelope type	[115]
Building type (flat, tower, both)	[116]
No. of elevators	[116]
Roof type	[116]
Type of public area (hall, corridor)	[116]
No. of pilotis	[116]

**Table 8:** Most commonly used performance metrics for deciding the soil shear strength and pre-project cost and duration.

Performance Metric Used	Usage Frequency	Reference
RMSE	10	[66,67,68,69,71,72,73,74,75,76]
Correlation coefficient (*R*)	9	[59,67,68,69,71,74,75,82,85]
MAPE	8	[59,66,79,80,81,82,83,84,85]
MAE	6	[68,69,71,73,74,76]
Coefficient of determination (*R^2^*)	3	[66,72,73]
VAF	2	[66,76]
AAE	2	[68,69]
Nash–Sutcliffe coefficient of efficiency	1	[68]
CRM	1	[72]
Cohesion equation	1	[74]

**Table 9:** Most commonly used AI techniques for predicting the soil shear strength and pre-project cost and duration.

AI Technique Used	Usage Frequency	Reference
ANN	16	[59,67,69,72,73,74,75,76,78,79,80,81,82,83,84,85]
Regression	5	[67,76,79,83]
SVM	3	[69,71,80]
Genetic algorithm	2	[78,79]
LSSVM	1	[66]
CSO	1	[66]
PNN	1	[64]
FNN	1	[68]
Dragon fly Algorithm	1	[73]
Whale optimization Algorithm	1	[73]
Invasive weed optimization	1	[73]
CBR	1	[79]
RBFNN	1	[59]
GRNN	1	[59]
MLP	1	[59]

## Data Availability

No new data were created or analyzed in this study. Data sharing is not applicable to this article.

## List of Acronyms

AAE	Absolute average error
AI	Artificial intelligence
ANN	Artificial neural network
BRNN	Bayesian regularization neural network
CBR	Case-based reasoning
CoD	Coefficient of determination
CPWD	Central Public Work Department
CRM	Coefficient of residual mass
CSO	Cuckoo search optimization
DENN	Differential evolution neural network
DL	Deep learning
FFNN	Feed-forward neural network
FNN	Functional neural network
GA	Genetic algorithm
IoT	Internet of things
KDA	Kernel discriminant analysis
LSSVM	Least square support vector machine
LSZ	Landslide susceptibility zonation
MAE	Maximum average error
MAPE	Mean absolute percentage error
ML	Machine learning
MLP	Multilayer perceptron
MRA	Multiple regression analysis
PNN	Probabilistic neural network
RBFNN	Radial basis function neural network
RMSE	Root-mean-square error
RNN	Recurrent neural network
SVM	Support vector machine
VAF	Variance accounted for
